# Green-synthesized silver nanoparticles from *Camellia sinensis*: mechanistic insights into phenolic-mediated multifunctional biological activities

**DOI:** 10.1186/s12870-025-07881-0

**Published:** 2025-12-09

**Authors:** Adem Demir

**Affiliations:** Central Research Laboratory, Recep Tayyip Erdoğan University, Rize, 53100 Türkiye

**Keywords:** Camellia sinensis, Silver nanoparticles, HPLC-DAD, Enzyme inhibition, Anti-inflammatory, Antibacterial activity, Molecular docking

## Abstract

**Background:**

Green synthesis of silver nanoparticles (AgNPs) using plant extracts provides an eco-friendly route that bridges phytochemistry with nanobiotechnology. *Camellia sinensis*, rich in polyphenols, serves as an effective reducing and stabilizing agent in this process.Ethanolic extracts of *C. sinensis* leaves were characterized for phenolic composition by HPLC-DAD and utilized for AgNP synthesis (CS-AgNPs). The nanoparticles were characterized by UV-Vis, FTIR, XRD, SEM, EDX, and DLS. Biological properties were assessed through antioxidant (DPPH, ABTS), antibacterial (Gram-positive and Gram-negative strains), anti-inflammatory (BSA denaturation), and enzyme inhibitory (urease, α-glucosidase) assays, supported by molecular docking.

**Results:**

EGCG was the dominant phenolic compound (54.8 ± 2.8 mg/g). CS-AgNPs displayed strong antioxidant (IC_50_: 28.78 µg/mL for DPPH), anti-inflammatory (IC_50_: 66.78 µg/mL), antibacterial (16.78–25.31 mm inhibition zones), and enzyme inhibitory activities (urease IC_50_: 20.6 µg/mL; α-glucosidase IC_50_: 140.9 µg/mL). Docking analysis confirmed the strong binding affinity of EGCG with target enzymes (−12.386 and −10.129 kcal/mol).

**Conclusions:**

This study highlights the dual role of *C. sinensis* phenolics in nanoparticle formation and bioactivity modulation, offering mechanistic insight into phenolic–metal interactions. Future work will focus on cytotoxicity and in vivo evaluations to validate the biomedical potential of CS-AgNPs.

**Supplementary Information:**

The online version contains supplementary material available at 10.1186/s12870-025-07881-0.

## Background

Phyto-derived silver nanoparticles (AgNPs) have received significant attention due to their tunable physicochemical properties and broad applications in medicine, pharmaceuticals, catalysis, and environmental science [1–5]. Although several chemical and physical methods for nanoparticle synthesis have been developed, many rely on toxic reagents, high energy input, and expensive procedures. In contrast, green synthesis offers an eco-friendly, cost-effective, and scalable route for nanoparticle production by employing plant metabolites as both reducing and stabilizing agents [6–11]. *Camellia sinensis* is a rich natural source of polyphenols, flavonoids, terpenoids, and alkaloids that contribute to its diverse biological activities. Catechins, particularly epigallocatechin gallate (EGCG), are the most abundant bioactives in tea and are known for their strong antioxidant, anti-inflammatory, anticancer, and enzyme inhibitory effects [12, 13]. These biomolecules not only provide health benefits but also play a vital role in the reduction of silver ions (Ag^+^) to metallic AgNPs and the stabilization of nanoparticle surfaces, leading to enhanced biological functionality [14, 15]. In recent years, the use of medicinal and edible plants for green nanoparticle synthesis has expanded rapidly, aiming to improve the antioxidant, antimicrobial, and anti-inflammatory properties of the resulting materials and to explore their potential in pharmaceutical and biomedical applications [16–19]. Recent studies have advanced plant-mediated silver nanoparticle synthesis by elucidating phenolic-mediated reduction, metal–ligand chelation, and capping mechanisms that link nanoparticle structure with multifunctional biological activity [20–22]. These investigations also demonstrate that drug-loaded or compositionally tuned AgNPs can exhibit superior antibacterial, antioxidant, and enzyme inhibitory properties, largely influenced by surface functionalization and phytochemical capping layers [23–25]. Studies on tea-based AgNPs have demonstrated promising antioxidant and antimicrobial properties; however, most of these works remain limited to basic physicochemical characterization and lack mechanistic or computational insight [26–32]. The present study addresses this gap by employing a comprehensive in vitro and in silico approach to elucidate the mechanistic basis of phenolic-mediated AgNP formation and to evaluate their multifunctional biological activities. Specifically, *C. sinensis* leaf extract was used for the green synthesis of AgNPs, followed by detailed physicochemical characterization and biological assessments, including antioxidant, antibacterial, anti-inflammatory, and enzyme inhibitory analyses. Molecular docking was further employed to understand the interaction mechanisms between dominant phenolics and target enzymes. This integrated approach provides new mechanistic insights into the relationship between phytochemical chelation and biological function, thereby highlighting the biomedical potential of C. sinensis-derived AgNPs. A schematic overview of the overall study design and workflow is provided in the Supplementary Material (Scheme S1) file.

## Materials and methods

### Plant and chemical reagents

*Camellia sinensis* (L.) Kuntze leaves were obtained from a tea garden in Rize, Türkiye. The specimen identification was performed by Dr. Esra Demir from the Recep Tayyip Erdogan University in Türkiye. A voucher specimen was deposited in the Herbarium of the Department of Biology (RTEU: 1253). All reagents used in the experiments were purchased from Sigma-Aldrich. Silver nitrate (AgNO_3_) salt and catechin powder (99% purity) were obtained from Sigma-Aldrich Chemical Co. and were used without further purification. Purified water was obtained from Human Corporation water purification system.

### Extraction of phenolic compounds in *Camellia sinensis*

A total of 10 g of green tea powder was extracted with 100 mL of ethanol at 40 °C for eight hours using a magnetic stirrer. To enhance the extraction efficiency of phenolic compounds, the mixture underwent ultrasonic-assisted extraction for one hour in a 40 kHz, 200 W ultrasonic bath. After extraction, the mixture was filtered through Whatman No. 1 filter paper. The resulting extract was then used for HPLC-DAD analysis and green synthesis of silver nanoparticles.

### Determination of phenolic compounds by HPLC-DAD

Phenolic compounds were analyzed using a Shimadzu Prominence LC-20 A HPLC-DAD system (Shimadzu, Japan) equipped with a reverse-phase C18 column (150 mm × 4.6 mm, 5 μm; GL Sciences, Japan). The mobile phases were: A (2% acetic acid in water) and B (acetonitrile: water, 70:30, v/v), with a gradient elution from 5% to 80% B over 30 min. The injection volume was 20 µL, flow rate 1.0 mL/min, and column temperature 30 °C. Quantification was performed using external calibration curves (0.5–25 mg/L) of catechin, epigallocatechin, epicatechin, epigallocatechin gallate, epicatechin gallate, gallic acid, and caffeine.

### Green synthesis of silver nanoparticles from *Camellia sinensis*

Silver nanoparticles (AgNPs) were synthesized by mixing 10 mL of 1 mM AgNO_3_ solution with 10 mL of green tea extract and stirring the mixture at room temperature in the dark for 16 h. The pH of the reaction mixture was adjusted to 8.0 using 0.1 M NaOH. A color change from green to dark brown indicated AgNP formation. The nanoparticles were collected by centrifugation at 10,000 rpm for 30 min, dried at 50 °C, and used for characterization and biological activity assays. The yield of AgNPs was 68.5% (w/w) based on the initial AgNO_3_ concentration.

### Characterization of AgNPs

The characterization of nanoparticles from *C. sinensis* leaf extract was carried out using a variety of analytical techniques, including UV-vis spectroscopy (Thermo Fisher Scientific, Multiskan GO), X-ray diffractometry (XRD, Rigaku-SmartLab), Fourier transform infrared spectroscopy (FTIR, Perkin Elmer Spectrum 100), dynamic light scattering (DLS, Malvern Zetasizer Nano), scanning electron microscopy (SEM, JEOL JSM 6610), and energy dispersive X-ray spectroscopy (EDS, Oxford Instruments Inca X-act) attached to the SEM.

### α-glucosidase inhibitory activity

The α-glucosidase inhibitory activity of the samples was determined with slight modifications to the method described by Wu et al. [33]. Briefly, 40 µL of α-glucosidase enzyme solution (0.7 U/mL) and 40 µL of the sample solution at various concentrations were pre-incubated at 37 °C for 10 min. The reaction was initiated by the addition of 200 µL of 1.0 mM p-nitrophenyl-α-D-glucopyranoside (pNPG), prepared in 50 mM phosphate buffer (pH 6.8). The mixture was incubated at 37 °C for an additional 10 min, after which the absorbance was measured at 405 nm using a microplate reader. The inhibition percentage was determined using Eq. (1), and IC_50_ values were calculated accordingly. Acarbose was used as a reference inhibitor for comparison.

1 $$\mathrm{Inhibition}\;(\%)\;=\;\left[1\;-\;\left({\mathrm A}_{\mathrm{sample}}\;/\;{\mathrm A}_{\mathrm{control}}\right)\right]\;\times\;100$$

### Urease inhibitory potential

Urease inhibitory activity was determined using the indophenol method with minor modifications based on the procedure of Weatherburn [34]. In a 96-well plate, 30 µL of urease enzyme solution (5 U/mL) and 30 µL of test samples (1–10 mg/mL) were mixed with 75 µL of 100 mM urea and incubated at room temperature for 10 min. Subsequently, 60 µL of reagent A (0.005% w/v sodium nitroprusside and 1% w/v phenol) and 105 µL of reagent B (0.1% NaOCl and 0.5% w/v NaOH) were added. After an additional 10 min incubation, absorbance was measured at 625 nm using a microplate reader (Molecular Devices, USA). Inhibition percentages were calculated using Eq. (1) and IC_50_ values were determined. Thiourea was used as the reference inhibitor.

### Anti‑inflammatory potential of Ag‑NPs

Anti-inflammatory activity was assessed via inhibition of protein denaturation assay using the method described by Ruiz-Ruiz et al., with slight modifications [35]. Briefly, test tubes containing 50 µL of different concentrations of the standard (diclofenac sodium), samples and methanol as a control were prepared. Bovine serum albumin (BSA) (450 µL, 5% w/v) was added to these tubes and incubated at 37 °C for 15 min, followed by heating at 70 °C for 3 min. After cooling, 2.5 mL of phosphate-buffered saline (pH 6.3) was added to each tube. The absorbance of the solutions was measured at a wavelength of 660 nm using a UV-visible spectrophotometer (T80 model PG Instrument, UK). The IC_50_ was determined using Eq. (1), which represents the concentration required to inhibit 50% of BSA denaturation.

###  Radical scavenging activity of Ag‑NPs

Radical scavenging activity was determined using the DPPH and ABTS methods. DPPH radical scavenging capacity was performed according to the method of Demir et al. [36]. Briefly, 0.15 mL of sample was mixed with 0.15 mL of freshly prepared 100 µM DPPH and incubated for 40 min in the dark and the absorbance was measured at 517 nm. Gallic acid and quercetin were used as references. For ABTS assay, 50 µL of the sample was added with 250 µL of ABTS radical solution and kept in the dark for 30 min and the absorbance was measured at 734 nm using a microplate reader (Multiskan GO, Thermo Fisher Scientific, USA). The results are expressed as SC_50_ values using Eq. (1). These values represent the minimum concentrations of compounds required to scavenge 50% of the DPPH and ABTS radicals. All assays were performed in triplicate.

### Antibacterial activity of Ag‑NPs

The antibacterial activity of the samples was determined by the disc diffusion method with minor modifications of the method used by Karsli et al. [37]. In this experiment, a sample concentration of 1 mg/ml was used. Gram-negative bacterial strains (*Escherichia coli* ATCC 25922, *Salmonella enterica* ATCC 13076, *Aeromonas hydrophila* ATCC 7966 and *Aeromonas sobria* ATCC 43979) and gram-positive strain (*Staphylococcus aureus* ATCC 25923) were used in this experiment. Tetracycline and sulfamethoxazole were used as reference standards and ethanol as a negative control. Results were obtained by measuring the diameter of the inhibition zones.

### Protocol of docking study

To investigate the binding interactions and mechanism of urease and glucosidase activity, the molecular docking simulations were employed between selected enzymes and determined 7 compounds of *C. sinensis* leaf extract, utilizing the Schrödinger Maestro software suite [38]. The entire process encompasses three primary stages, beginning with protein preparation. For this, the crystal structures of the target enzymes, Helicobacter pylori urease (PDB ID: 6ZJA) and alpha-glucosidase-I (PDB ID: 4J5T), are retrieved from the Protein Data Bank (PDB) server [39, 40]. Subsequently, these enzyme structures are prepared using the Protein Preparation Wizard within the Schrödinger Maestro software suite. The determined 7 compounds of *C. sinensis* leaf extract were downloaded from PubChem server and prepared with LigPrep module of Maestro User Interface. All prepared ligands were docked with the active sites of the respective receptor structures. The induced-fit docking (IFD) method, using the standard Glide/XP protocol included in the Schrödinger software suite, was used for docking process. To validate the docking protocol, the co-crystallized ligands are re-docked into their respective binding sites. The details of docking protocol were given in Supplementary Material file.

### Statistical analysis

All experiments were performed in triplicate, and the results are presented as mean ± standard deviation (SD). Data normality was assessed using the Shapiro–Wilk test, and homogeneity of variances was verified by Levene’s test. Statistical differences among groups were analyzed by one-way analysis of variance (ANOVA), followed by Tukey’s post hoc test for multiple comparisons. Differences were considered statistically significant at *p* < 0.05. All analyses were carried out using SPSS software, version 18.0 (IBM Corp., Armonk, NY, USA).

## Results

### HPLC-DAD analysis of *Camellia sinensis* leaf extract

**Fig. 1:**
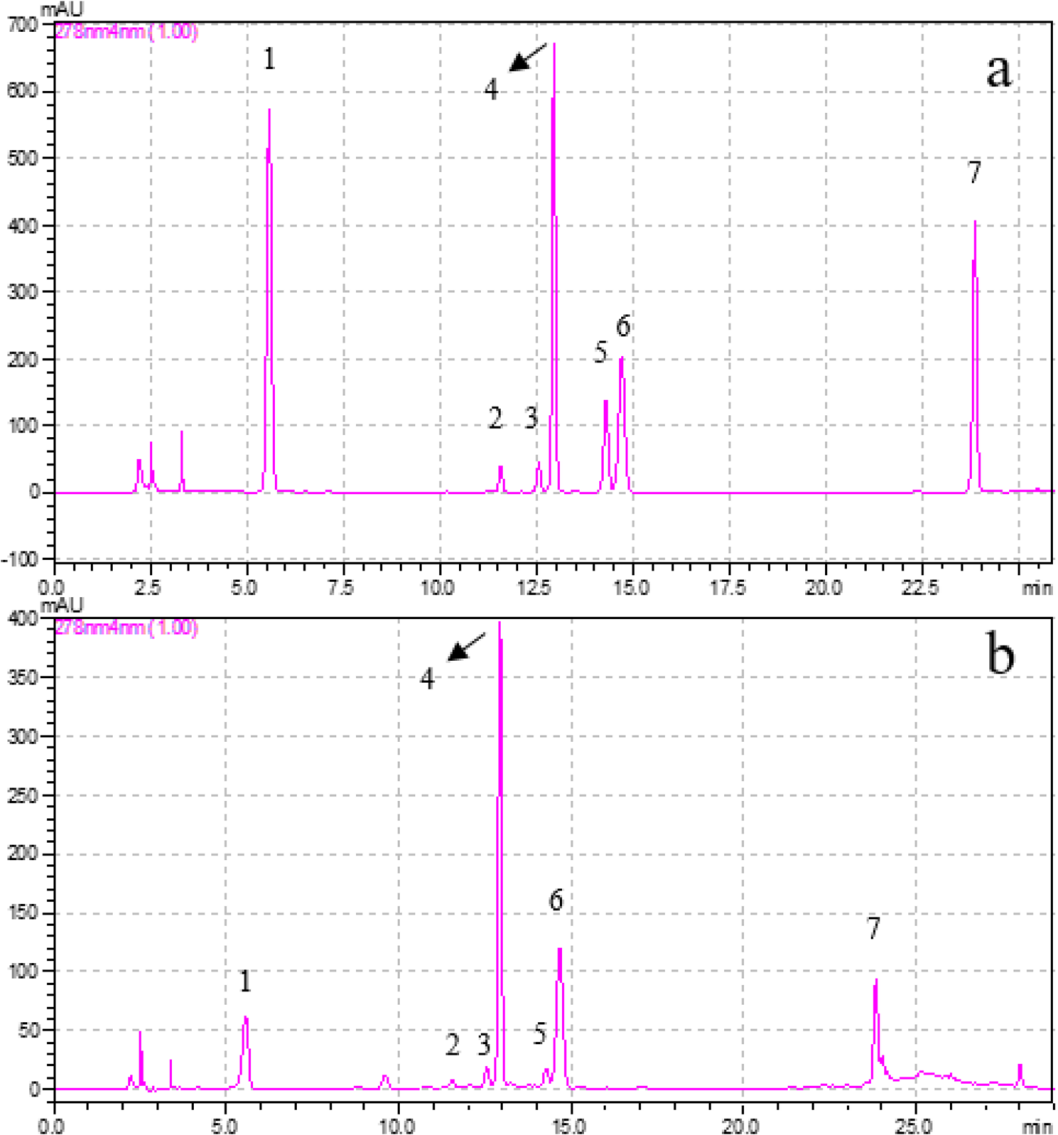
HPLC-DAD chromatograms recorded at 278 nm: **a** Standards mixture solution, **b** Green tea leaf extract 1: Gallic acid, 2: EGC, 3: C, 4: Caffeine, 5: EC, 6: EGCG, 7: ECG (R^2^: 0.995–0.999)

The phenolic compounds found in the ethanol extract of green tea leaves were quantitatively analyzed using an HPLC-DAD system. First, calibration curves were created using the external standard method for seven phenolic compounds: gallic acid, epigallocatechin (EGC), catechin (C), caffeine, epicatechin (EC), epigallocatechin gallate (EGCG), and epicatechin gallate (ECG). All calibration curves showed excellent linearity, with correlation coefficients (*R*^*2*^) ranging from 0.995 to 0.999, confirming the accuracy and reliability of quantification. The analysis revealed that the highest concentration was detected in EGCG at 54.8 ± 2.8 µg/mg, followed by caffeine at 25.8 ± 1.3 µg/mg and ECG at 20.3 ± 1.2 µg/mg. The amounts of the seven phenolic compounds are given in Table 1, and the standard mixture and sample chromatograms are presented in Fig. 1. These findings are consistent with earlier reports identifying EGCG as the predominant catechin in *Camellia sinensis* extracts from various geographical sources [41–45]. The concentrations of EGCG, ECG, and caffeine observed here closely matches that described by Zhao et al. and Lee et al., despite methodological differences in extraction and quantification [46, 47]. Such compositional agreement supports the reliability of the extraction procedure and confirms that the ethanolic extract preserved the characteristic catechin profile of green tea, providing a chemically rich precursor for the biosynthesis of silver nanoparticles.

**Table 1:** HPLC-DAD analysis of the ethanolic extract of *Camellia sinensis* leaf

Phenolic component	Green Tea Leaf (mg/g)
Gallic acid	7.9 ± 0.4
Epigallocatechin (EGC)	3.9 ± 0.1
Catechin (C)	3.8 ± 0.2
Caffeine	25.8 ± 1.3
Epicatechin (EC)	4.2 ± 0.3
Epigallocatechin gallate (EGCG)	54.8 ± 2.8
Epicatechin gallate(ECG)	20.3 ± 1.2

Data are expressed as mean ± SD (*n* = 3)

### Physical characterization of AgNPs

**Fig. 2:**
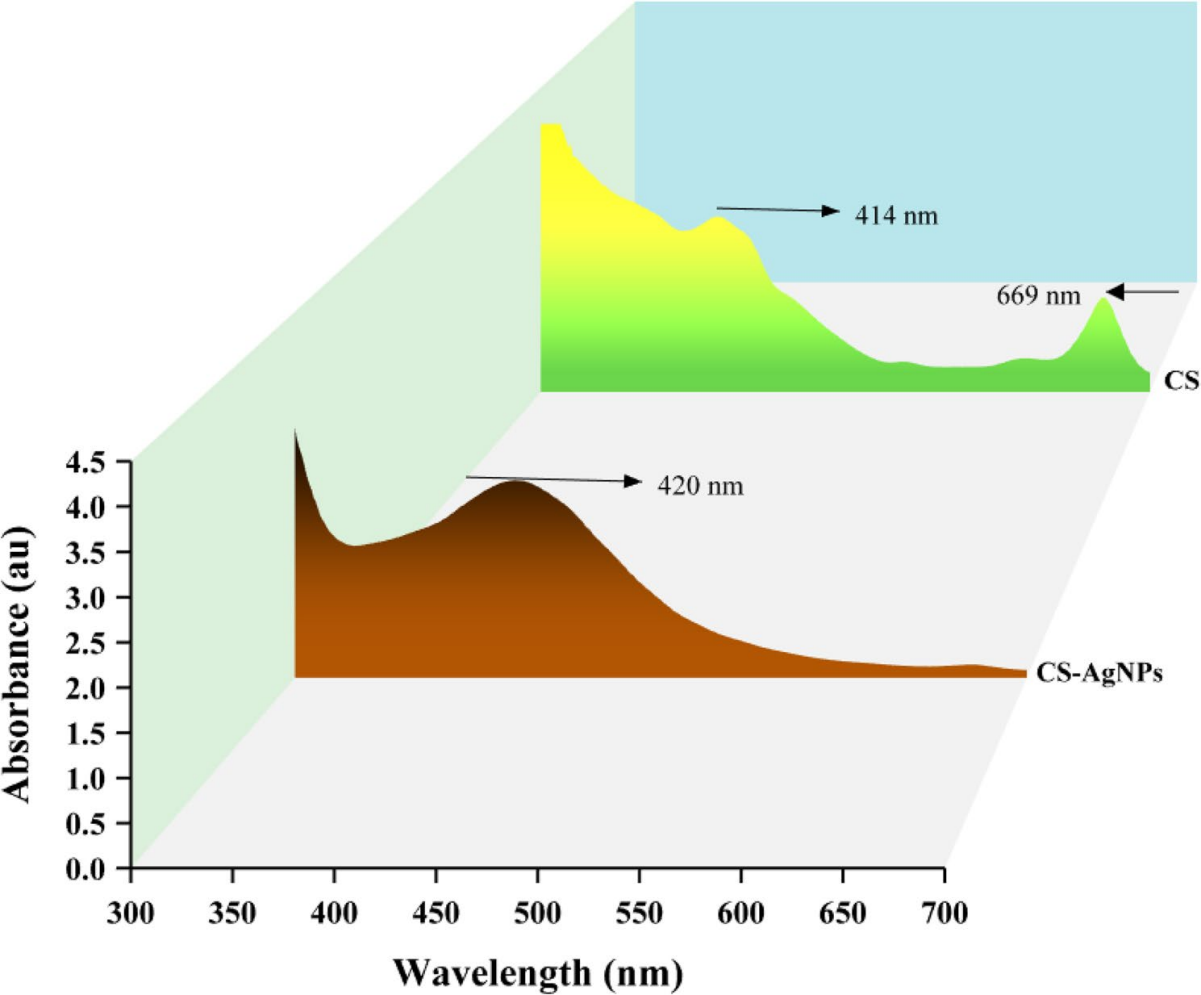
UV–Vis spectra of *Camellia sinensis* extract (CS) and CS-AgNPs

**Fig. 3:**
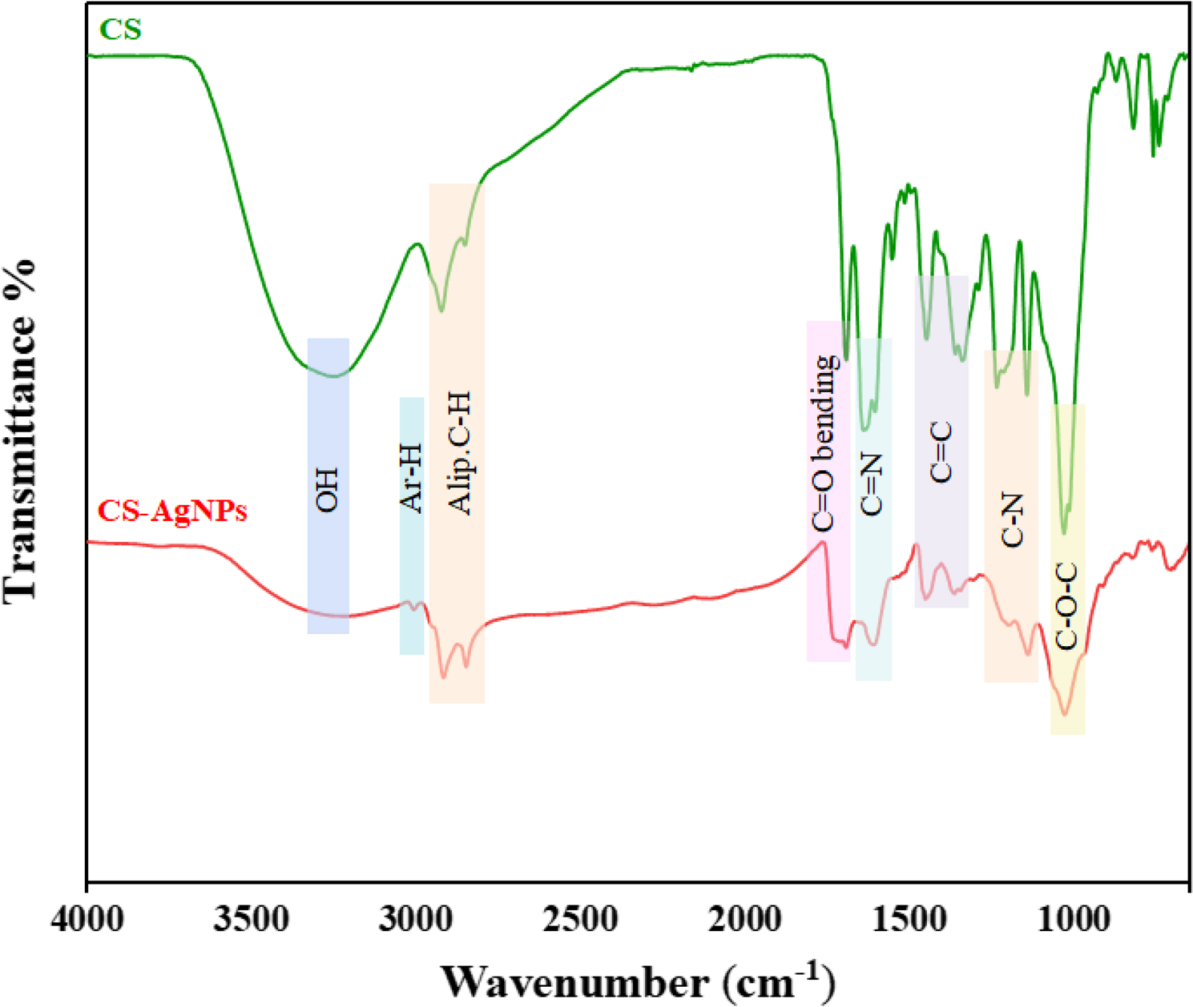
FT-IR spectra of *Camellia sinensis* extract (CS) and CS-AgNPs

The UV–Vis spectrum of AgNPs is based on a phenomenon commonly referred to as surface plasmon resonance (SPR) [48]. The green tea extract used as the starting material in this synthesis did not exhibit any plasmonic peak in the same region but showed two absorption peaks at 414 nm and 669 nm, corresponding to *n–π* and *π–π* transitions originating from functional organic groups [49]. After nanoparticle formation, a characteristic SPR peak appeared at 420 nm (Fig. 2), confirming the successful synthesis of AgNPs. This band arises from electron oscillation across the dielectric medium and nanoparticle interface [50]. Furthermore, the disappearance of the 669 nm peak in the AgNP spectrum indicates the consumption or transformation of the functional chromophores during the reduction of Ag^+^ ions. The FTIR spectrum was used to elucidate the structural and chemical properties of the nanoparticles and their interactions with organic molecules [51]. In the FTIR spectrum of the green tea extract, a broad band was observed at 3274 cm^−1^, corresponding to the O-H stretching vibrations of catechin derivatives, while aliphatic C–H stretching bands appeared at 2925–2857 cm^−1^. The C = O, C = N, and C = C stretching vibrations were observed at 1698, 1646, and 1453 cm^−1^, respectively. After nanoparticle formation, several of these bands became less intense and slightly broadened, with a minor shift of the O-H stretching band from 3274 to 3265 cm^−1^ and the C = O band from 1698 to 1689 cm^−1^. These spectral changes suggest that hydroxyl and carbonyl groups were involved in the reduction and capping of Ag^+^ ions, confirming that polyphenolic compounds acted as both reducing and stabilizing agents for the synthesized AgNPs (Fig. 3).

**Fig. 4:**
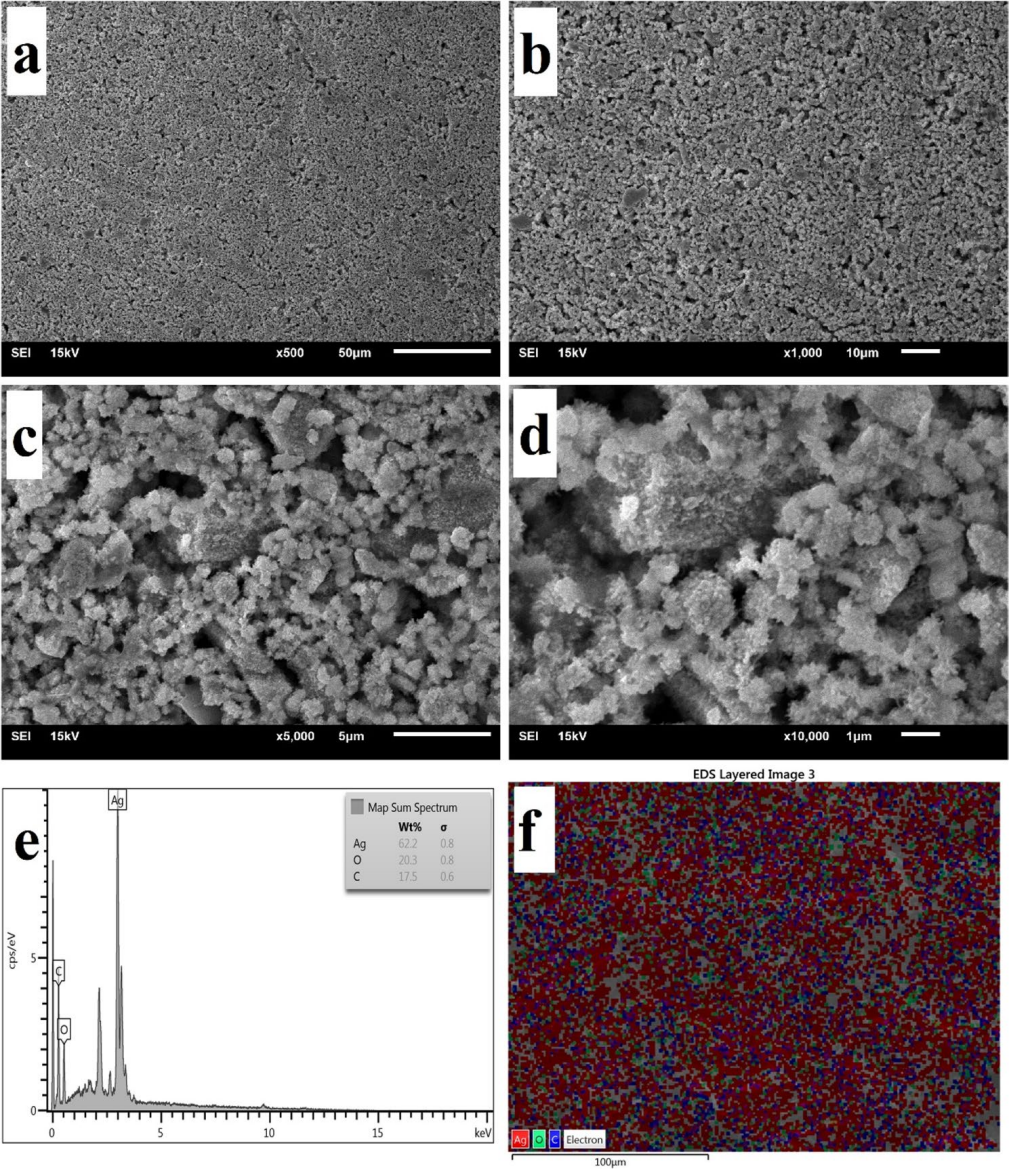
SEM images (**a**–**d**) and EDX spectra (**e**, **f**) of CS-AgNPs showing nanoparticle morphology and elemental composition

**Fig. 5:**
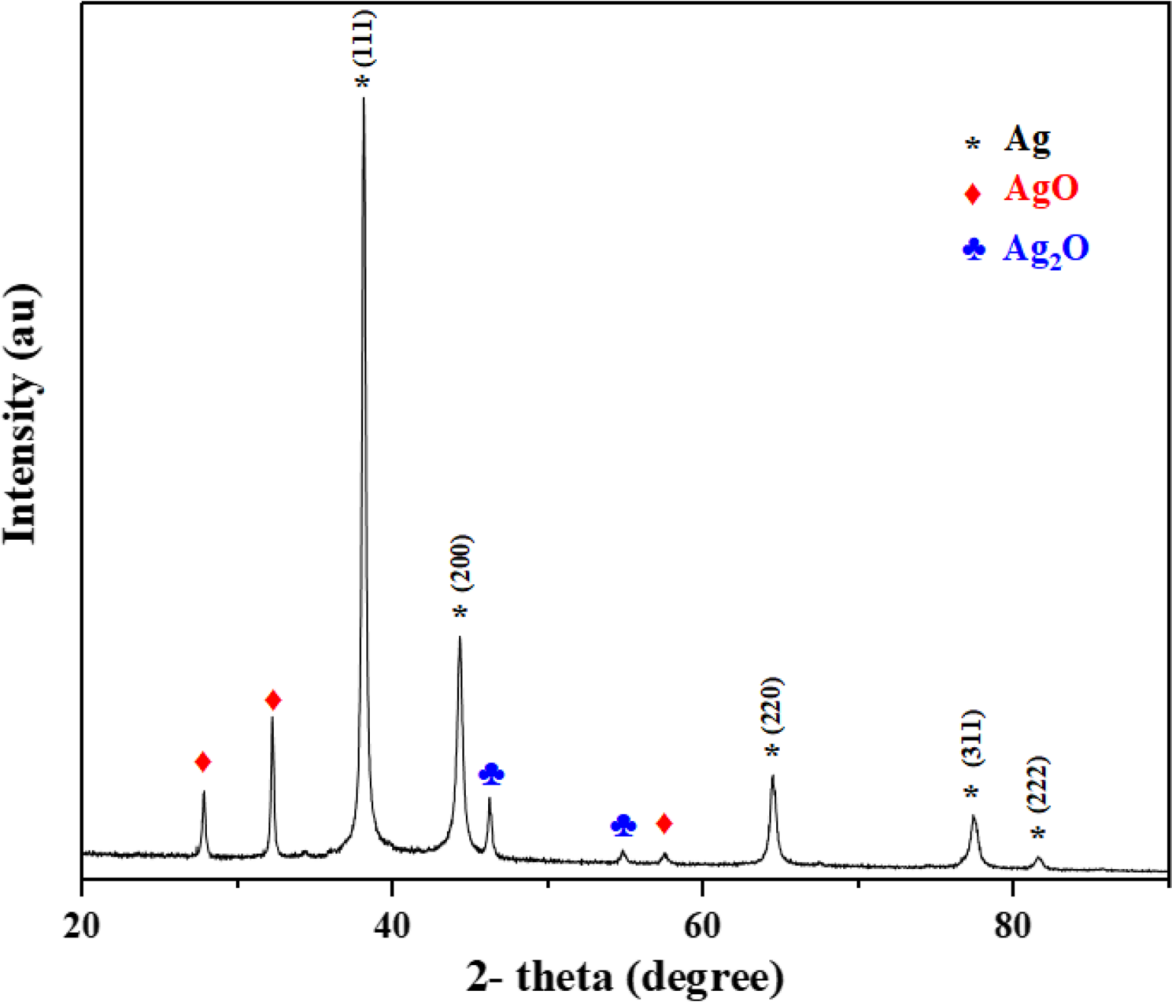
XRD pattern of CS-AgNPs showing crystalline structure

**Fig. 6:**
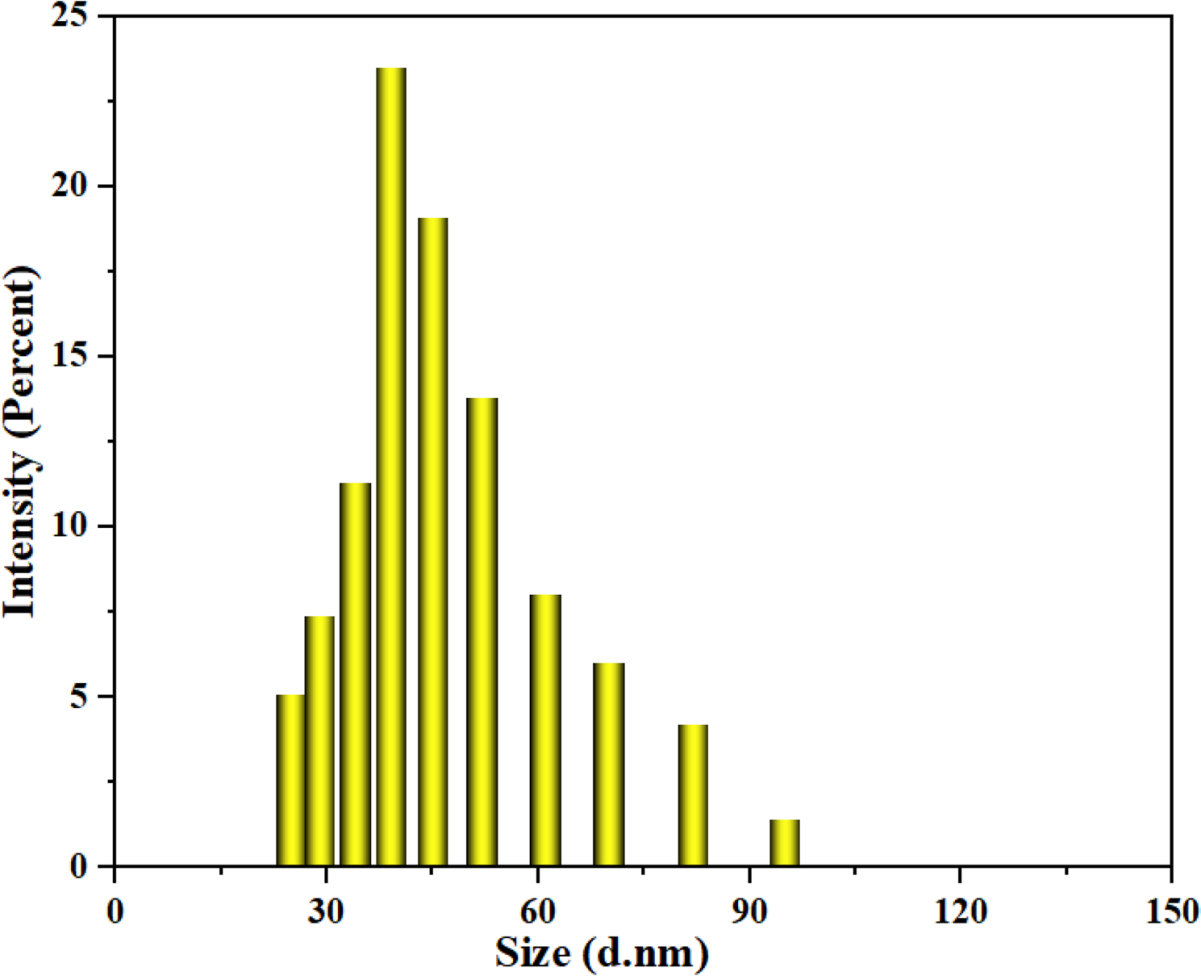
Particle size distribution of CS-AgNPs by DLS analysis

**Fig. 7:**
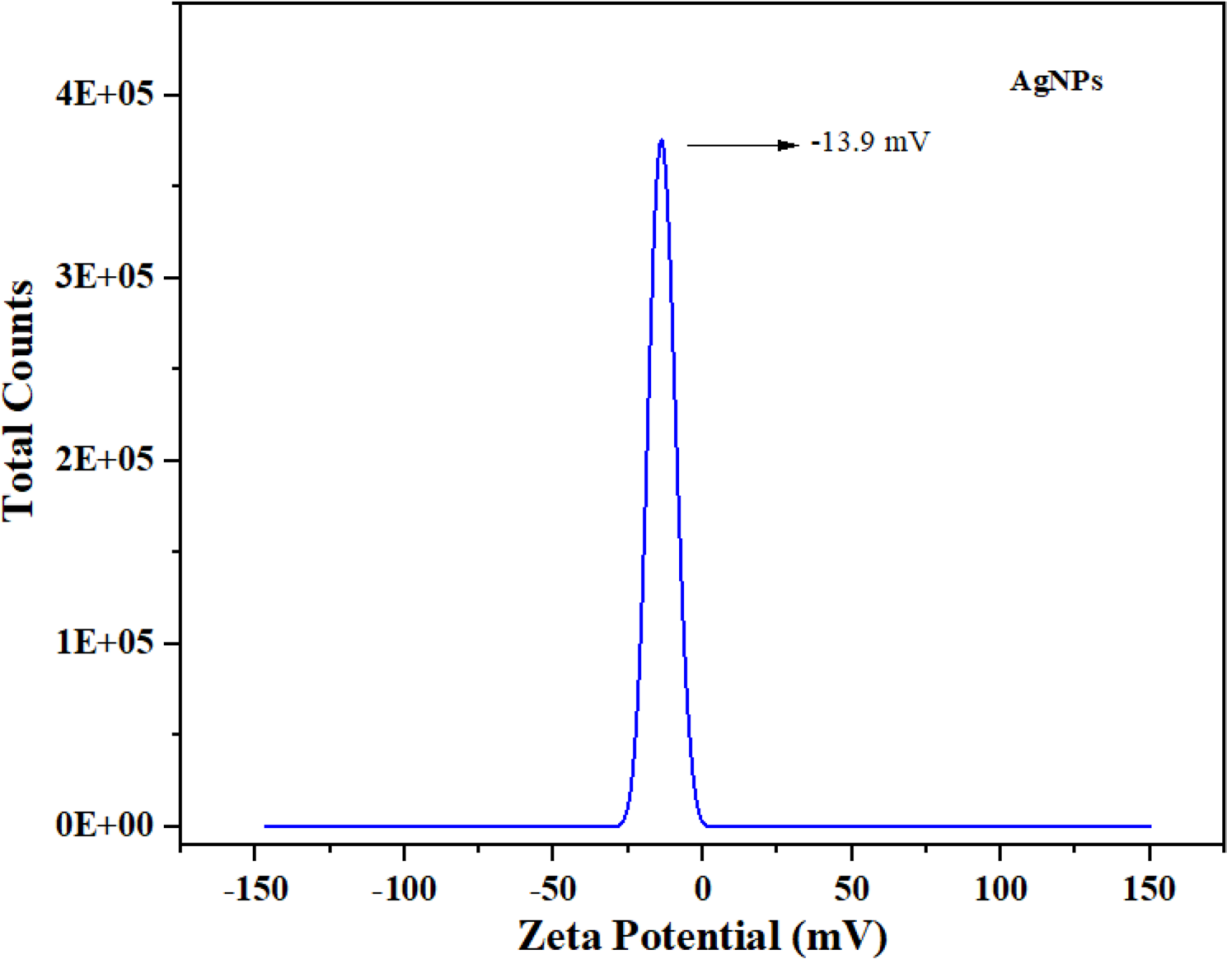
Zeta potential spectrum of synthesized CS-AgNPs

The SEM images confirmed the spherical morphology and uniform distribution of the biosynthesized AgNPs (Fig. 4). The XRD patterns of AgNPs (Fig. 5) displayed distinct diffraction peaks at 2θ = 38.06° (111), 44.26° (200), 64.41° (220), 77.37° (311), and 81.49° (222), corresponding to the face-centered cubic structure of metallic silver (JCPDS No. 03–065−2871). Additional minor peaks at 27.80°, 32.20°, 46.20°, 54.78°, and 57.39° were attributed to AgO and Ag_2_O phases, indicating partial surface oxidation. The average crystallite size of AgNPs was calculated as 38 nm using the Debye–Scherrer equation. Dynamic light scattering (DLS) analysis (Fig. 6) revealed that the hydrodynamic particle size of AgNPs ranged from 34 to 52 nm, which is slightly larger than the crystallite size obtained from XRD. This difference arises because DLS measures the hydrodynamic diameter including the metallic core, organic capping layer, and solvated shell whereas XRD reflects only the crystalline domain [52, 53]. The particle size measured by DLS is therefore considered as the total size of the metallic core plus the organic ligand shell and associated solvent layer [54]. The zeta potential of the AgNPs was − 13.9 mV (Fig. 7), indicating a moderately stable colloidal dispersion due to electrostatic repulsion among particles. A zeta potential between − 30 mV and + 30 mV generally indicates a stable nanoparticle system [55]. This value also confirms the presence of anionic functional groups on the nanoparticle surface.

### α-glucosidase inhibition activity

**Fig. 8:**
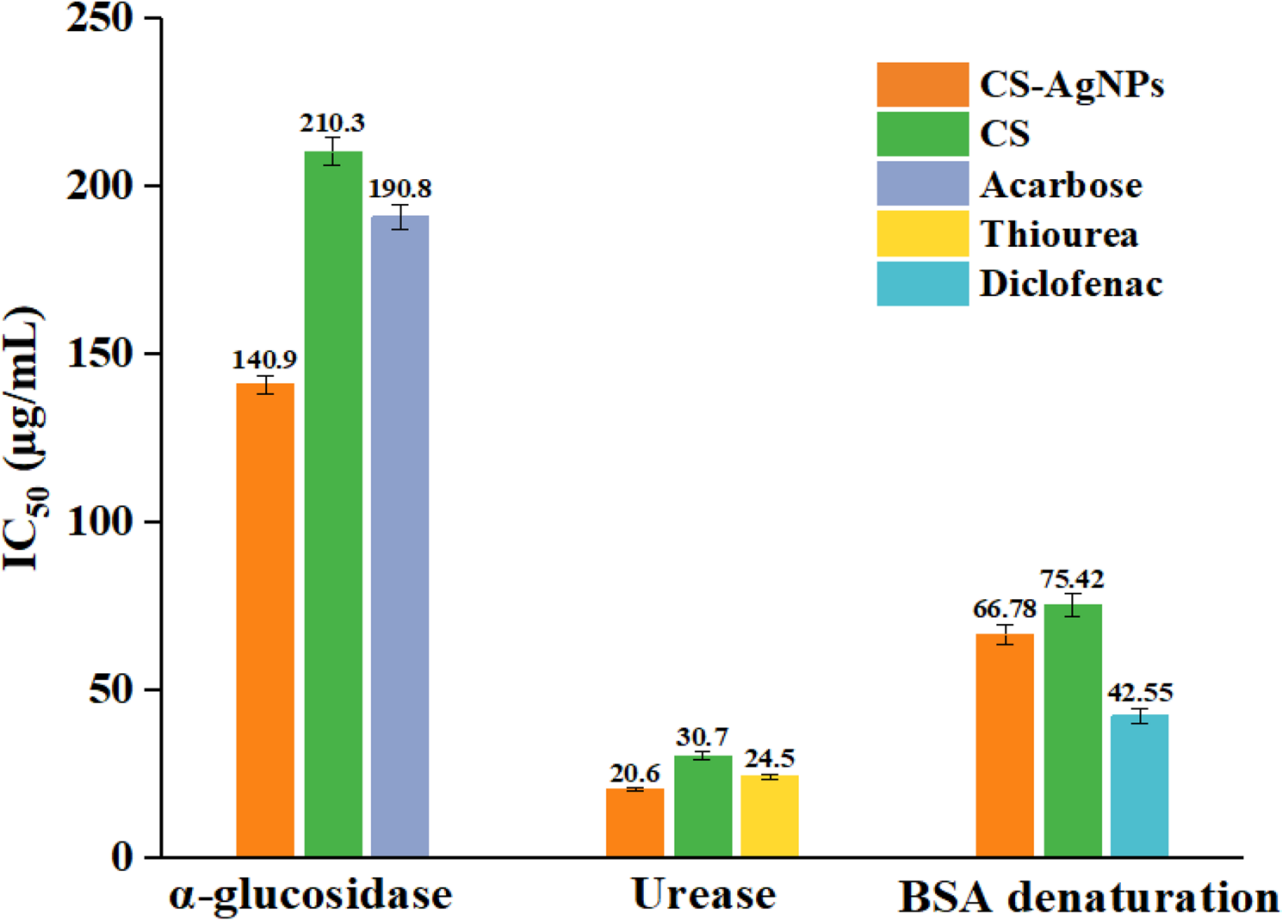
α–glucosidase, urease and anti-inflammatory activities of CS, CS-AgNPs, and standards

The α-glucosidase inhibition activity of green tea extract (CS), its silver nanoparticles (CS-AgNPs), and acarbose is presented in Table 2; Fig. 8. The CS-AgNPs exhibited significantly higher α-glucosidase inhibitory activity compared to both CS and acarbose (*p* < 0.05, one-way ANOVA followed by Tukey’s post hoc test). The IC_50_ values for α-glucosidase inhibition were 140.9 ± 2.8, 210.3 ± 4.1, and 190.8 ± 3.8 µg/mL for CS-AgNPs, CS, and acarbose, respectively. These results indicate that the biosynthesized CS-AgNPs exhibit stronger α-glucosidase inhibitory activity than both the plant extract and the standard drug acarbose. Similarly, Esposito et al. found that green tea showed a much higher inhibitory effect than acarbose (IC_50_: 72 µg/mL, 1250 µg/mL), and Orita et al. reported that ethanolic green tea extracts were more effective than aqueous ones in both rat and human cell models [56, 57]. Kamiyama et al. also observed potent inhibition by green tea, with IC_50_ values of 45 µg/mL and 190 µg/mL against maltase and sucrase, respectively, while Li et al. showed that green tea extract (IC_50_: 0.4 mg/mL) was nearly three times more active than acarbose (IC_50_: 1.10 mg/mL) [43, 58].

**Table 2:** α-glucosidase and urease inhibition capacity of CS and CS-AgNPs

Sample	α-glucosidase IC_50_ (µg/mL)	Urease IC_50_ (µg/mL)
CS	210.3 ± 4.1	30.7 ± 1.1^c^
CS-AgNPs	140.9 ± 2.7	20.6 ± 0.4^a^
Acarbose	190.8 ± 3.8	
Thiourea		24.5 ± 0.7^b^

Data are expressed as mean ± SD (*n* = 3)

Different superscript letters (a-c) within a column indicate significant differences (*p* < 0.001, one-way ANOVA, Tukey’s post hoc test)

### Urease inhibition activity

The effect of CS, CS-AgNPs, and thiourea on urease activity is presented in Table 2; Fig. 8. The IC_50_ values obtained from different concentrations were 20.6 ± 0.4, 30.7 ± 1.1, and 24.5 ± 0.7 µg/mL for CS-AgNPs, CS, and thiourea, respectively. The urease inhibitory activity of CS-AgNPs was significantly higher than that of CS and thiourea (*p* < 0.05, one-way ANOVA followed by Tukey’s post hoc test). Matsubara et al. showed that green tea extract inhibited *H. pylori* urease with an IC_50_ of 13 µg/mL and reduced gastritis development in vivo, while Hassani et al. reported that *Camellia sinensis* methanol: water extracts completely suppressed ureA and ureB expression at 2.5–3.5 mg/mL [59, 60]. Similarly, AgNPs synthesized from *L. anatolica* root extract exhibited stronger urease inhibition (IC_50_: 3.32 µg/mL) than both the crude extract (7.94 µg/mL) and thiourea (6.03 µg/mL) [61].

### Anti‑inflammatory potential of Ag‑NPs

The anti-inflammatory properties of CS and CS-AgNPs were evaluated using the BSA denaturation inhibition test (Table 3; Fig. 8). The IC₅₀ values were 66.78 ± 3.1 µg/mL for CS-AgNPs, 75.42 ± 3.4 µg/mL for CS extract, and 42.55 ± 2.2 µg/mL for diclofenac sodium. CS-AgNPs showed significantly higher inhibitory activity than the extract (*p* < 0.05, one-way ANOVA, Tukey’s post hoc test). Similar anti-inflammatory effects have been reported for other green-synthesized nanoparticles. Nayel et al. found that *Lactuca anatolica* root extract, AgNPs, and diclofenac inhibited BSA denaturation by 65.15%, 57.58%, and 40.91% at 125 µg/mL, respectively [61]. Rajakumar reported that ZnO nanoparticles synthesized from *Andrographis paniculata* leaf extract showed higher activity (IC_50_: 66.78 µg/mL) than the extract alone (IC_50_: 75.42 µg/mL) [62]. Similarly, Thatoi et al. demonstrated that Ag and ZnO nanoparticles from mangrove plants exhibited IC_50_ values between 63.29 and 114.88 µg/mL [63].

**Table 3:** Radical scavenging and anti‑inflammatory activities of CS and CS-AgNPs

	DPPH SC_50_ (µg/mL)	ABTS SC_50_ (µg/mL)	BSA Denaturation Inhibition IC_50_ (µg/mL)
CS	35.31±2.1^d^	51.71±2.1^d^	75.42 ±3.4^c^
CS-AgNPs	28.78±1.3^c^	44.82±1.9^c^	66.78 ±3.1^b^
Quercetin	15.51±0.8^b^	29.17±1.3^b^	
Trolox	12.74±0.7^a^	24.51±0.7^a^	
Diclofenac sodium			42.55 ±2.2^a^

Data are expressed as mean ± SD (n = 3)

Different superscript letters (a-d) within a column indicate significant differences (*p* < 0.001, one-way ANOVA, Tukey’s post hoc test)

### Radical scavenging activities of Ag‑NPs

**Fig. 9:**
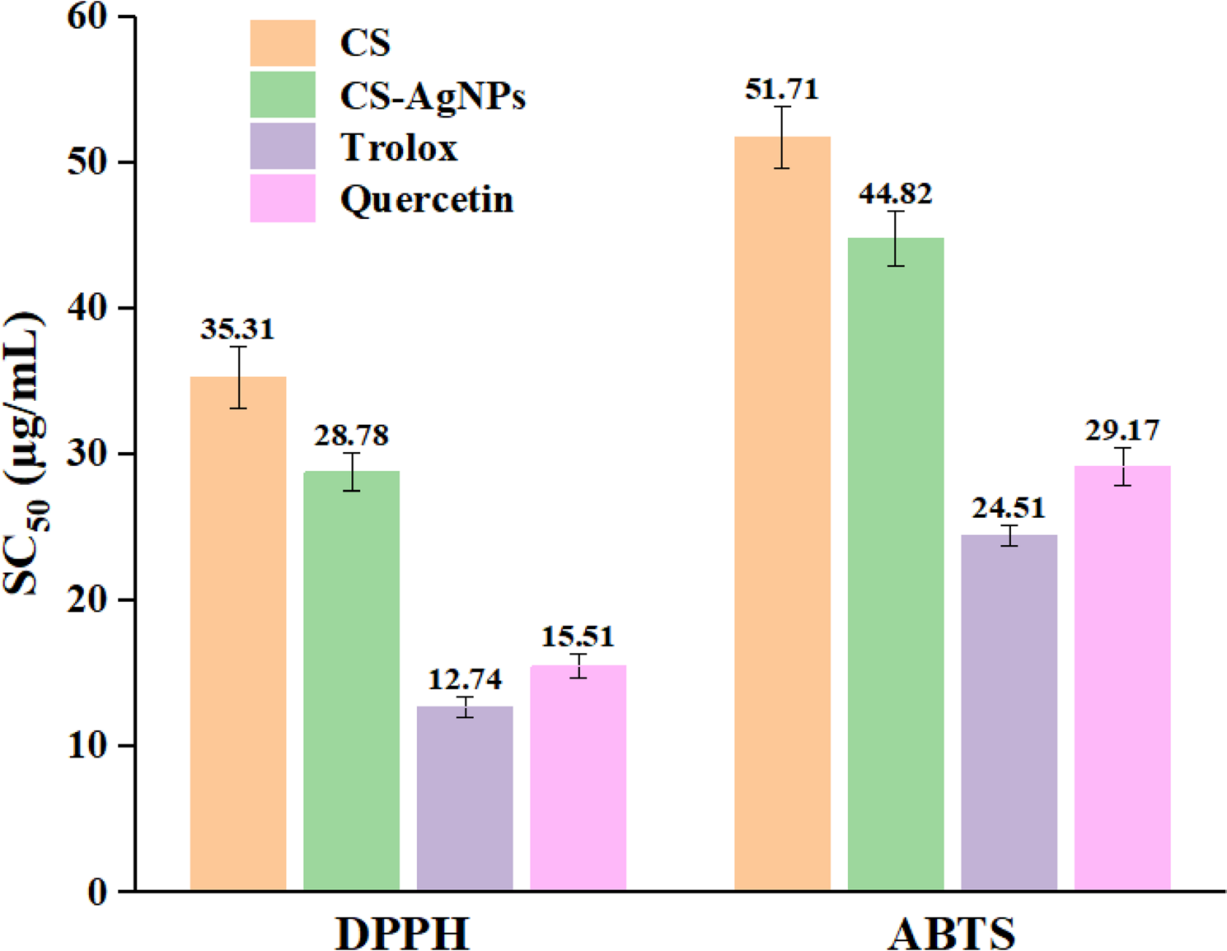
DPPH and ABTS radical scavenging activities of CS, CS-AgNPs, and standards

The SC_50_ values indicating the antioxidant activities of CS-AgNPs, CS extract, and reference standards are presented in Table 3; Fig. 9. CS-AgNPs demonstrated strong DPPH radical scavenging activity with an SC_50_ value of 28.78 µg/mL and ABTS⁺ cation scavenging activity with an SC_50_ value of 44.82 µg/mL. The radical scavenging activity of CS-AgNPs was significantly higher than that of the *C. sinensis* extract (*p* < 0.05, one-way ANOVA followed by Tukey’s post hoc test). Mobaraki et al. found that green tea–derived AgNPs exhibited DPPH and ABTS scavenging activities comparable to those of rutin, while the plant extract showed lower activity [32]. Selvan et al. reported SC_50_ values of 6.88 ± 1.08 µg/mL and 6.89 ± 0.66 µg/mL for ABTS and DPPH radical scavenging, respectively, in AgNPs synthesized from green tea, indicating strong antioxidant potential [64]. Likewise, Li et al. demonstrated that green tea polyphenol complexes exhibited SC_50_ values of 16.24 ± 0.99 µg/mL (DPPH) and 5.13 ± 0.17 µg/mL (ABTS) [58].

### Antibacterial potential of Ag‑NPs

The antimicrobial potential of CS-AgNPs against Gram-negative and Gram-positive bacteria was evaluated using the disc diffusion method, and the results are presented in Table 4. CS-AgNPs exhibited antibacterial activity against all tested strains, with inhibition zones ranging from 16.78 to 25.31 mm. The mean inhibition zones of CS-AgNPs were significantly larger than those of the CS extract (*p* < 0.05, one-way ANOVA followed by Tukey’s post hoc test). Bergal et al. observed that AgNPs synthesized from green tea leaves produced inhibition zones of 7–22 mm against *E. coli* and *S. aureus*, depending on synthesis conditions [30]. Singh et al. reported strong antibacterial activity of AgNPs from Indian green tea leaf extract, with MIC values of 0.16 mg/mL for *P. aeruginosa* and 0.08 mg/mL for *S. aureus* [31]. Likewise, Asghar et al. found that AgNPs synthesized from green and black tea showed inhibition zones of 19–20 mm against *S. aureus*, surpassing Cu- and Fe-based nanoparticles [65].

**Table 4:** Antibacterial potential of CS and CS-AgNPs

Sample	*E. coli*	*S. aureus*	*S. enterica*	*A. hydrophila*	*A. sobria*
CS	22.80±0.34^a^	23.86±0.17^a^	22.64±0.32^a^	15.95±0.08^a^	22.88±0.67^a^
CS-AgNPs	24.10±0.20^b^	24.73±0.35^b^	24.06±0.43^b^	16.78±0.08^b^	25.31±0.96^b^
Tetracycline	22.79±0.13^a^	28.18±0.32^c^	25.37±0.41^c^	23.53±0.05^c^	23.59±0.12^a^
Sulfamethoxozol	25.61±0.07^c^	31.24±0.38^d^	29.36±0.13^d^	25.04±0.09^d^	27.56±0.07^c^

Data are expressed as mean ± SD (n = 3)

Different superscript letters(a-d) within each column indicate significant differences (*p* < 0.001, one-way ANOVA, Tukey’s post hoc test)

### Molecular docking analyses

A molecular docking study was performed to assess the predicted binding affinities of seven phenolic compounds identified in *Camellia sinensis* leaf extract against two crucial enzyme targets: *α-*glucosidase I and *Helicobacter Pylori* Urease. The simulations were conducted using the Induced Fit Protocol (IFD) within the Schrödinger Maestro program. Docking scores, where more negative values signify stronger predicted binding affinities, were utilized to evaluate the interactions. To verify the reliability of the docking protocol, the co-crystallized ligand of *H. pylori* urease (PDB ID: 6ZJA) was re-docked into its native active site, yielding an RMSD value of 1.304 Å. An RMSD below 2 Å is generally considered acceptable, confirming the robustness of the docking protocol applied in this study.

The docking analysis revealed a range of predicted binding affinities for the phenolic compounds against both enzyme targets. The docking scores of these compounds are given in Table 5. For α-glucosidase I, Epicatechin gallate (ECG) exhibited the most favorable predicted binding with a score of −10.246, closely followed by Epigallocatechingallate (EGCG) at −10.129. Epigallocatechin (EGC) also showed a strong predicted affinity with a score of −9.461. Conversely, Caffeine displayed the weakest predicted interaction with a score of −4.666, indicating its lowest affinity among the tested compounds for this enzyme. Gallic acid and Catechin (C) showed moderate affinities, with scores of −8.103 and − 8.085, respectively.

**Table 5:** Docking scores of phenolic compounds in *Camellia sinensis* leaf extract against α-glucosidase and urease enzymes (kcal/mol)

Phenolic component	α-glucosidase I	*Helicobacter Pylori* Urease
Gallic acid	−8.103	−6.726
Epigallocatechin (EGC)	−9.461	−9.915
Catechin (C)	−8.085	−9.991
Caffeine	−4.666	−4.432
Epicatechin (EC)	−8.775	−9.28
Epigallocatechingallate (EGCG)	−10.129	−12.386
Epicatechin gallate(ECG)	−10.246	−11.021

Regarding *Helicobacter Pylori* Urease, EGCG was identified as the most potent predicted binder, achieving a significantly strong score of −12.386. This was followed by ECG at −11.021, and Catechin (C) at −9.991, indicating robust interactions with this enzyme as well. Consistent with its performance against *α-*glucosidase I, Caffeine again exhibited the lowest predicted affinity with a score of −4.432.

A comparative analysis across both enzymes indicated that EGCG demonstrated the highest overall predicted affinity, particularly for *Helicobacter Pylori* Urease. While ECG also exhibited strong binding to both targets, EGCG’s affinity for Urease was notably higher than its affinity for α-glucosidase I. Catechin (C) also showed a distinct preference for Urease, with a score of −9.991 compared to −8.085 for α-glucosidase I.

**Fig. 10:**
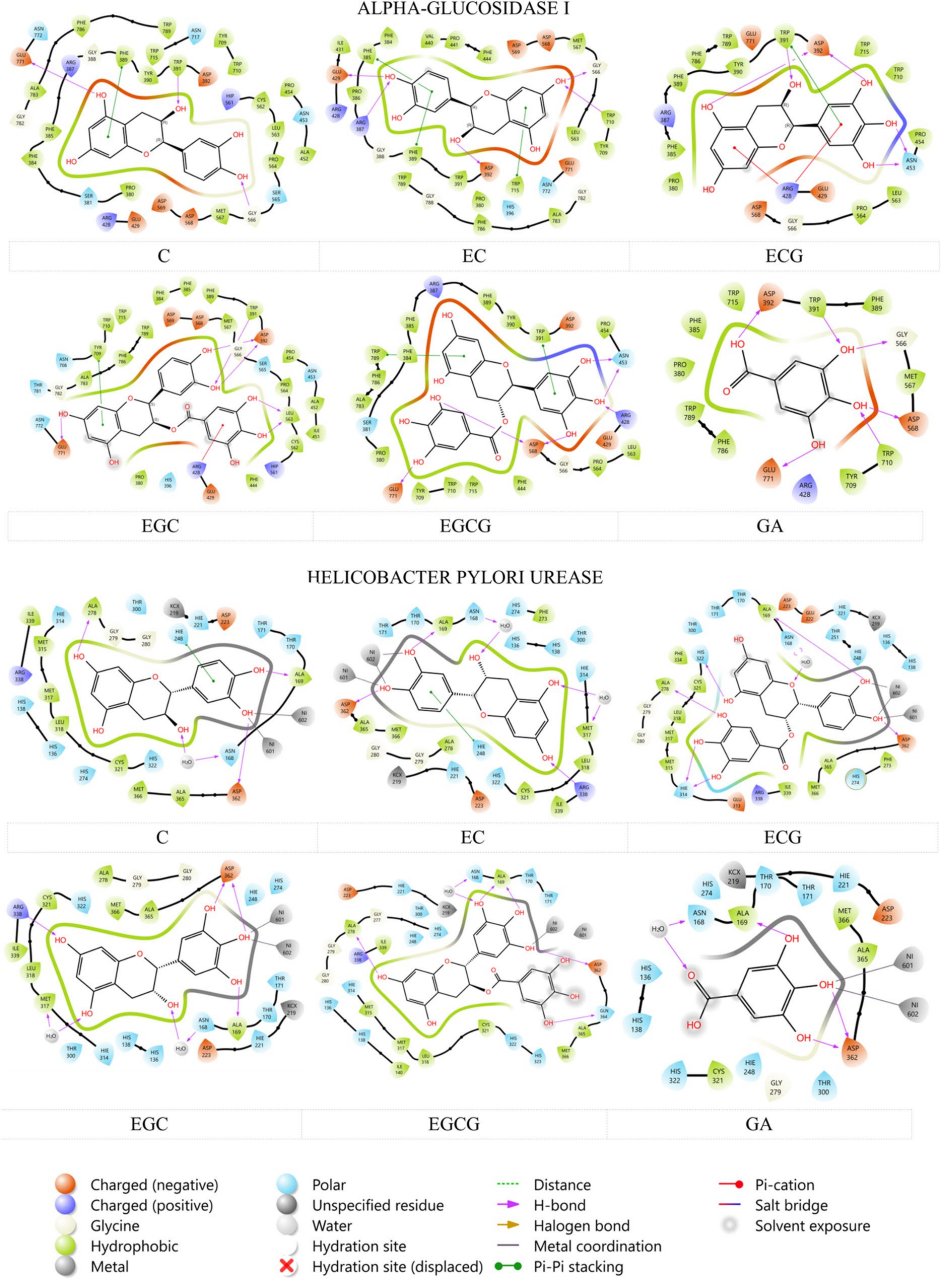
Two-dimensional representation of phenolic compound interactions within the active sites of *Helicobacter pylori* urease and α-glucosidase I

The localization of compounds with the highest in silico binding scores in the respective protein active sites was investigated in detail. In this context, the interactions between the enzyme α-glucosidase I and its ECG ligand were analyzed using the presented 2D interaction diagram in Fig. 10. The docking pose of ECG, identified as one of the compounds exhibiting the most favorable predicted binding affinities for *α-*glucosidase I, was examined in detail within the enzyme’s active site. This detailed analysis was conducted to elucidate the specific molecular interactions contributing to the stability of the ECG-α-glucosidase I complex.

Hydrogen bonding interactions were observed to play a significant role in anchoring ECG within the binding pocket. Multiple hydroxyl groups of the gallate moiety were found to form direct hydrogen bonds with the amino acid residue Leu 563. Additionally, the epicatechin moiety of ECG was shown to form direct hydrogen bonds with Trp 391, Asp 392 and Glu 771. Pi-pi stacking interaction was also noted as contributing to the ligand’s stable conformation within the active site. The benzene ring of the gallate moiety was observed to engage in pi-cation interaction with Arg 428. Concurrently, the epicatechin ring was found to participate in a pi-pi stacking interaction with Tyr 709. Hydrophobic interactions were extensively distributed throughout the binding pocket, providing substantial stabilization to the complex.

A meticulous examination of Epigallocatechin Gallate (EGCG)’s docking pose within the active site of *Helicobacter pylori* urease was undertaken, following its prediction as the compound with the most favorable binding affinity. Critical hydrogen bonding interactions were found to anchor EGCG within the urease binding pocket extensively. Direct hydrogen bonds were formed by the hydroxyl group of EGCG with the Gln 364, Ala 169, Ala 278 and Asp 362 residues. Beyond direct contacts, water molecules were observed to bridge additional hydrogen bonds between a hydroxyl group and Ala 168 residue. Metal coordination was also observed between the hydroxyl group and the Ni^+ 2^ ions, which are crucial for enzyme activity. Similarly, significant hydrophobic interactions also played a role in stabilizing the ligand’s positioning. These contacts encompass a wide range of hydrophobic amino acid residues surrounding the EGCG molecule, including Ile 339, Met 315, Met 317, Leu 318, Ile 140, Cys 321, Ala 365, and Met 366. The complementary hydrophobic nature of certain regions in the active site has been observed to facilitate strong interactions with the aromatic components of EGCG.

Overall, considering the interactions of other phenolic compounds in Fig. 10, the complex docking positions adopted by the compounds in the active sites of both *Helicobacter pylori* urease and α-glucosidase I enzymes revealed a complex network of both direct and water-bridged hydrogen bonds supported by extensive hydrophobic contacts. This diverse array of non-covalent interactions suggests that these compounds have potent inhibitory potential against this enzyme, providing the molecular basis for the predicted high binding affinity for both enzymes.

## Discussion

Numerous medicinal plants have been reported as effective biological resources for the green synthesis of nanoparticles, including *Pachygone laurifolia* [4], *Helianthemum lippii* [9], *Phoenix dactylifera* [10], *Olea europaea* [24], *Eucalyptus globulus* [66], *Falcaria vulgaris* [67], *Curcuma longa* (turmeric) [68], *Chenopodium album* [69], *Anacardium occidentale* [70], *Naringi crenulata* [71], *Ocimum sanctum* [72], *Cinnamomum camphora* [73], *Rubus fruticosus* [74], *Stevia rebaudiana* [75], *Glycyrrhiza glabra* [76], *Camellia sinensis* [26, 30, 32], *Hibiscus sabdariffa* [77], *Thymus vulgaris* [78], and *Camellia sinensis*-derived black tea [79]. Various plant parts such as leaves, roots, flowers, stems, seeds, and latex are utilized in nanoparticle synthesis due to their rich content of bioactive phytochemicals [25, 80].

In this study, ethanol was used as a green solvent and ultrasonic-assisted extraction was employed to obtain phenolic compounds. Ethanol is low in toxicity, biodegradable, and approved by the U.S. FDA for food applications, while ultrasonic extraction enhances efficiency and reduces solvent use, making it both eco-friendly and effective for phenolic recovery [81, 82].

High-performance liquid chromatography (HPLC) is widely recognized as a reliable and precise method for identifying phenolic compounds due to its high separation efficiency and analytical accuracy [83]. In this study, HPLC-DAD analysis confirmed that the ethanolic extract of *Camellia sinensis* contained seven main phenolic constituents, with EGCG being the dominant compound, followed by caffeine and ECG. The results indicate that the extraction method effectively preserved the characteristic catechin composition of green tea. This phenolic profile is significant, as catechins with strong redox and metal-chelating properties play a central role in the antioxidant, enzyme-inhibitory, and anti-inflammatory activities observed in the synthesized CS-AgNPs.

Inhibition of α-glucosidase plays a crucial role in managing postprandial hyperglycemia associated with type 2 diabetes [84, 85]. The findings of this study demonstrate that CS-AgNPs exhibit stronger inhibitory activity compared to both the crude extract and the standard drug acarbose, indicating their potential as natural enzyme inhibitors. The enhanced activity may arise from the synergistic effects of silver and catechins, particularly EGCG and ECG, which can interact with enzyme active sites through hydrogen bonding and π–π stacking interactions, facilitating stable enzyme–inhibitor complexes. Similar results in α-glucosidase inhibition have also been reported for ciprofloxacin-loaded AgNPs (CIP@AgNPs), which achieved 68% inhibition at 60 µg/mL, combining strong antidiabetic and anti-inflammatory effects with enhanced antibacterial activity against multidrug-resistant *S. aureus* and *E. coli*. Likewise, Mg–Ag-doped ZnO nanoparticles synthesized from *Phoenix dactylifera* seed extract displayed superior α-amylase and α-glucosidase inhibition compared to undoped ZnO NPs, confirming that Ag incorporation enhances surface reactivity and facilitates electron transfer processes [10, 21]. These comparative findings suggest that the improved enzyme-inhibitory efficiency of CS-AgNPs is governed by similar metal–ligand coordination and surface redox mechanisms.

Similarly, the urease inhibition results revealed superior activity for CS-AgNPs compared to both the extract and thiourea. Since elevated urease activity and ammonia formation contribute to *H. pylori*-associated gastric disorders, the inhibition observed here is pharmacologically relevant [86]. As supported by literature, catechins can inhibit urease by coordinating with Ni^2+^ ions and disrupting the ureA/ureB subunits [60]. The formation of AgNPs likely amplifies this effect by improving enzyme–nanoparticle contact and modulating Ag^+^ ion availability. However, as quantitative assessment of Ag^+^ release was not conducted, its precise contribution remains uncertain. Previous reports suggest that partial dissolution of Ag^+^ from biogenic AgNPs enhances enzyme inhibition through synergistic interactions between ionic silver and phytochemical capping agents. Therefore, the inhibitory activity of CS-AgNPs likely results from the combined effect of direct nanoparticle–enzyme interaction and limited ionic silver contribution [20, 87]. At the molecular level, the inhibitory mechanism can be attributed to chelation and metal–ligand coordination processes. Polyphenolic compounds such as EGCG and ECG possess ortho-dihydroxyl and carbonyl groups capable of donating electrons to Ag^+^ ions, forming stable Ag-O coordination bonds during nanoparticle formation. These interactions not only promote uniform nucleation and surface stabilization but also mimic similar coordination behaviors in biological systems. For instance, catechin hydroxyl groups and AgNP surfaces can interact with metal centers (Ni^2+^ in urease), disrupting metalloenzyme activity, while hydrogen bonding and π–π stacking with residues such as Asp, Glu, and Trp enhance α-glucosidase inhibition. Collectively, these findings indicate that enzyme inhibition primarily arises from direct competitive binding of catechins at catalytic sites, supported by synergistic coordination with the nanoparticle surface. This interaction model aligns with recent findings demonstrating that the surface chemistry of biogenic AgNPs significantly modulates their biological activity [61]. In particular, phenolic-mediated electron transfer and Ag-O coordination influence the redox potential and adsorption dynamics at the enzyme interface, thereby enhancing catalytic site binding affinity. The ability of catechin-capped nanoparticles to control localized Ag^+^ ion availability further amplifies inhibitory potency while minimizing nonspecific interactions [88].

The anti-inflammatory potential of CS-AgNPs was demonstrated through the BSA denaturation inhibition assay, where nanoparticles exhibited higher inhibitory capacity than the extract. The suppression of protein denaturation reflects the ability to maintain protein integrity under stress, a hallmark of anti-inflammatory potential. The observed enhancement may result from synergistic interactions between the polyphenolic capping layer and denatured protein structures. Comparable results have been reported for ciprofloxacin-loaded AgNPs, which achieved 86.4% inhibition of albumin denaturation at 800 µg/mL, confirming the role of AgNPs in stabilizing protein conformations and reducing inflammatory damage [21]. Furthermore, *Olea europaea*-based Ag/Ag_2_O nanoparticles demonstrated significant in vivo anti-inflammatory and antioxidant effects by alleviating metribuzin-induced oxidative and hematological disturbances, highlighting the therapeutic promise of green-synthesized Ag nanostructures in inflammation management [24].

The antioxidant performance of CS-AgNPs, evaluated through DPPH and ABTS assays, was markedly higher than that of the *Camellia sinensis* extract. This improvement can be attributed to the nanoscale particle size, which enhances the reactive surface area for electron transfer, and the redox-active catechin layer, which facilitates efficient radical neutralization. The antioxidant mechanism likely involves both direct radical scavenging by catechins and electron transfer from the AgNP surface. Consistent with this, *Helianthemum lippii*-derived AgNPs showed potent DPPH radical scavenging activity (IC_50_: 3.16 µg/mL) and strong biological compatibility, while the same nanoparticles mitigated cadmium-induced hepatic toxicity by reducing oxidative stress and restoring normal liver architecture in vivo [9, 23]. These parallel findings reinforce that the combination of metallic silver and phenolic capping agents produces a synergistic redox interface, enhancing the antioxidant and protective potential of plant-derived AgNPs. Comparable mechanistic behavior has also been observed in other green-synthesized AgNP systems, where polyphenol capping layers mediate redox cycling and stabilize reactive intermediates [89]. Unlike these earlier reports, the present study integrates in vitro antioxidant assays with in silico analyses, directly linking the phenolic composition of *Camellia sinensis* to the observed redox activity.

CS-AgNPs exhibited pronounced antibacterial activity against both Gram-positive and Gram-negative bacteria, with inhibition zones exceeding those of the extract. This can be attributed to multiple mechanisms, including disruption of bacterial cell membranes, generation of reactive oxygen species, and Ag^+^ ion interaction with thiol-containing enzymes. Catechins further enhance this effect by damaging bacterial cell walls and interfering with metabolic enzymes, resulting in a synergistic antimicrobial response. Although previous studies generally report higher sensitivity in Gram-positive bacteria due to their simpler cell wall structure, the present study demonstrated stronger inhibition against certain Gram-negative strains such as *Aeromonas sobria* and *S. enterica* [90]. This difference may stem from strain-specific variations in cell wall composition, lipopolysaccharide content, and nanoparticle–surface interactions, as well as the unique phenolic composition of the *Camellia sinensis* extract. These results are consistent with recent studies showing that AgNPs synthesized using medicinal plant extracts exhibit strain-dependent antibacterial profiles influenced by nanoparticle size and surface functionality [91, 92]. However, the enhanced inhibition against *A. sobria* and *S. enterica* observed here suggests a distinctive synergistic effect between catechin capping and controlled Ag^+^ release, differentiating the CS-AgNP system from other biogenic AgNP formulations.

Molecular docking analysis supported the experimental findings by confirming strong binding affinities of EGCG and ECG to both α-glucosidase and *H. pylori* urease through hydrogen bonding, π–π stacking, and metal ion coordination, whereas caffeine showed minimal interaction, consistent with its lower biological activity. These results further validate that inhibition occurs mainly through direct and specific binding of catechins at enzyme catalytic sites rather than nonspecific surface adsorption [5, 93]. EGCG formed particularly extensive interactions with urease, including metal coordination with Ni^2+^ ions, suggesting high inhibitory potential [94].

While the present findings offer meaningful insights into the properties and biological potential of CS-AgNPs, further research is required to elucidate their underlying mechanisms and enhance their functional performance. Building on the current spectroscopic, biochemical, and molecular docking results, future studies should include in vivo and molecular-level validation to confirm the proposed mechanisms. Translating these mechanistic insights into biological models will be crucial for verifying their pharmacological relevance and advancing their potential biomedical applications. Investigating how factors such as nanoparticle size, morphology, and catechin composition affect bioactivity will help refine their design. Moreover, comprehensive evaluations of long-term stability, cytotoxicity, and biocompatibility under physiological conditions are essential to ensure the safe and effective application of CS-AgNPs in biomedical fields.

## Conclusions

HPLC-DAD analyses revealed that *Camellia sinensis* leaf extract is rich in catechins and galloylated derivatives, which serve as effective reducing and capping agents in the green synthesis of silver nanoparticles. The synthesized CS-AgNPs exhibited notable anti-inflammatory activity, with significant protein denaturation inhibition, suggesting their potential in managing inflammation-related conditions. Their strong antioxidant capacity, demonstrated through DPPH and ABTS assays, was attributed to polyphenolic constituents, particularly catechins and their derivatives. In antibacterial evaluations, CS-AgNPs displayed strong inhibitory effects against multidrug-resistant bacterial strains, especially *A. sobria* and *E. coli*, indicating potential applications in combating antibiotic-resistant infections. Moreover, the nanoparticles showed effective in vitro inhibition of *Helicobacter pylori* urease and α-glucosidase, suggesting possible therapeutic use in managing gastrointestinal infections and hyperglycemia. Molecular docking analyses supported these findings, demonstrating high binding affinities of galloylated catechins toward both enzymes. In future research, the cytotoxicity and biocompatibility of CS-AgNPs should be systematically evaluated through in vivo studies to confirm their biological safety and efficacy. In addition, the potential of catechin-stabilized silver nanoparticles as multifunctional platforms for targeted drug delivery and advanced pharmaceutical formulations deserves further exploration.

## Supplementary Information

Supplementary Material 1.

## Data Availability

The datasets supporting the results of this article are included in the article and Additional files.

## Abbreviations

CS: *Camellia sinensis*
HPLC-DAD: High performance liquid chromatography-diode-array detection
SEM-EDX: Scanning electron microscopy- energy dispersive X-ray spectroscopy
FT-IR: Fourier-transform infrared spectroscopy
XRD: X-Ray diffraction spectroscopy
DPPH: 1,1-diphenyl-2-picrylhydrazyl
ABTS: 2,2'-azino-bis(3-ethylbenzothiazoline-6-sulfonic acid)
BSA: Bovine serum albumin
IC, SC: Inhibition Concentration, Scavenging Concentration
