# Supplementary material for: Green-synthesized silver nanoparticles from Camellia sinensis: mechanistic insights into phenolic-mediated multifunctional biological activities
Source: BMC Plant Biol. 2025 Dec 9;25:1734. doi: 10.1186/s12870-025-07881-0 (PMC12743403; doi:10.1186/s12870-025-07881-0)
Supplement: Supplementary file 1 — Supplementary Material 1. [file 12870_2025_7881_MOESM1_ESM.docx]

**Green-Synthesized Silver Nanoparticles from *Camellia sinensis*: Mechanistic Insights into Phenolic-Mediated Multifunctional Biological Activities**

Adem Demir ^1,^*

^1^Central Research Laboratory, Recep Tayyip Erdoğan University, Rize 53100, Türkiye; *Correspondence: [adem.demir@erdogan.edu.tr](mailto:adem.demir@erdogan.edu.tr)

**SUPPLEMENTARY MATERIAL**


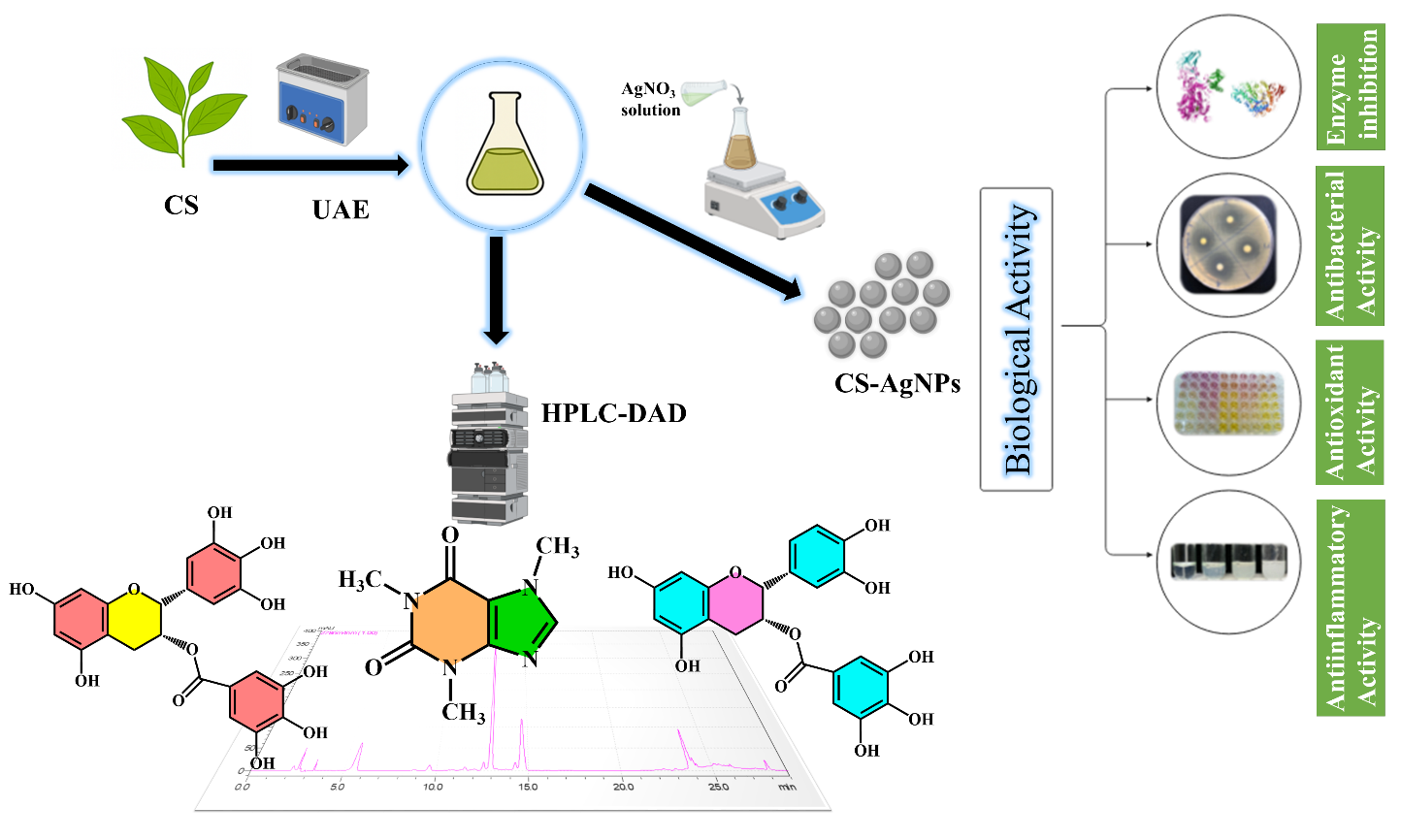


**Scheme S1.** Schematic workflow of the green synthesis, characterization, and biological evaluation of Camellia sinensis–derived silver nanoparticles

**Molecular Docking Protocol**

This section provides a comprehensive description of the molecular docking workflow employed in this study, performed using the Schrödinger Maestro software suite. The crystal structures of *Helicobacter pylori* urease (PDB ID: 6ZJA) and α-glucosidase-I (PDB ID: 4J5T) were downloaded from the Protein DataBank. Missing side chains and hydrogen atoms were added, and water molecules beyond 5 Å from the binding site were removed using the Protein Preparation Wizard. Protonation states were optimized at physiological pH (7.0 ± 0.2) with the PROPKA tool, and the protein structures were energy-minimized under the OPLS-2005 force field with an RMSD cut-off of 0.3 Å. Seven major compounds identified in *Camellia sinensis* leaf extract were obtained from PubChem and imported into Maestro. Ligand geometries were optimized using the LigPrep module with the OPLS-2005 force field, generating all relevant stereoisomers, ionization, tautomeric, and ring-conformational states. Ionization states at pH 7.0 (± 0.2) were predicted using the Epik module. The receptor grid was defined with a 20 Å radius around the catalytic binding site of each enzyme. Docking simulations were carried out using the Induced Fit Docking (IFD) protocol combined with the Glide/XP scoring function to accoun tfor both ligand and receptor flexibility. Binding-site coordinates were determined from the positions of the co-crystallized ligands in the enzyme structures. Validation of the docking protocol was achieved by re-docking the native ligands into their active sites, confirming RMSD values < 2 Å.
